# Irofulven and RSL3 synergistically target ferroptosis-related gene PTGR1 to treat head and neck squamous cell carcinoma

**DOI:** 10.1515/biol-2025-1332

**Published:** 2026-06-11

**Authors:** Xiyuan Li, Lu Lu, Shuyao Yang, Yinglin Su, Chenfei Liu, Jingxia Xu, Lezong Chen, Chunlei Dai, Xumiao Zhang, Zhenzhen Chou, Mingyuan Du, Qiaohong Lin, Jian Meng, Shiting Zhang, Xiao Zhang, Yuanbin Song, Ming Song

**Affiliations:** Department of Head and Neck Surgery, State Key Laboratory of Oncology in South China, Guangdong Provincial Clinical Research Center for Cancer, Sun Yat-sen University Cancer Center, Guangzhou, 510060, China; Department of Hematologic Oncology, Sun Yat-sen University Cancer Center, State Key Laboratory of Oncology in South China, Guangdong Provincial Clinical Research Center for Cancer, Guangzhou, 510060, China; Nanyang Second General Hospital, Nanyang, 473000, Henan, China; Department of Thyroid Surgery, Sun Yat-sen Memorial Hospital, Sun Yat-sen University, Guangzhou, 510120, China; Guangdong Province Key Laboratory of Malignant Tumor Epigenetics and Gene Regulation, Sun Yat-sen Memorial Hospital, Sun Yat-sen University, Guangzhou, 510120, China; Key Laboratory of Modern Preparation of TCM, Ministry of Education, Jiangxi University of TCM, Nanchang, 330004, China

**Keywords:** HNSCC, ferroptosis, PTGR1, irofulven, scRNA-seq

## Abstract

Head and neck squamous cell carcinoma (HNSCC) is an aggressive form of cancer characterized by significant fatality rates and unfavorable clinical outcomes. Ferroptosis serves as a crucial mechanism in controlling oncogenesis and tumor growth. However, the strategy targeting ferroptosis-related gene to predict and treat HNSCC is still not clear. The study was designed to assess the potential of ferroptosis as a biomarker and therapeutic target in HNSCC. A prognostic model based on ferroptosis-related genes was constructed using least absolute shrinkage and selection operator (LASSO). Multi-omic analysis revealed that the model was closely related to DNA methylation, DNA mutation and immune infiltration. HNSCC classification and the risk score in each classification were identified by analyzing single-cell RNA-sequencing (scRNA-seq) data. The results demonstrated that the risk score in malignant tumor classification is the highest, which is relative to the progression of HNSCC. Furthermore, through CellMiner database, we found the small molecular drug irofulven could target the risk score-related gene prostaglandin reductase 1 (PTGR1), which could inhibit the ferroptosis levels in HNSCC. Importantly, irofulven and ferroptosis inducer RSL3 exerted significant synergistic effects on HNSCC *in vitro* and *in vivo*. Altogether, this study offers a potential strategy for developing a combination therapy for targeting ferroptosis in the treatment of HNSCC.

## 1 Introduction

Head and neck squamous cell carcinoma (HNSCC) originates from the oral cavity, oropharynx, larynx, and hypopharynx [1]. Because most external organs are covered by the squamous epithelium, squamous cell carcinoma is even more dangerous. According to the 2020 Global Cancer Statistics Report, HNSCC ranks eighth among all malignant tumors worldwide, with approximately 840,000 new cases annually, and its incidence is projected to reach nearly one million cases by 2030 [2]. Localized HNSCC is classified as oral, oropharyngeal, laryngeal, hypopharyngeal, and nasopharyngeal carcinomas [3]. The main methods for the diagnosis of HNSCC are clinical imaging, pathological analysis, and qualitative local HNSCC [4], 5]. At present, HNSCC is usually treated with surgery, radiotherapy, chemotherapy, and the recently emerged immune and targeted therapy [6]. Surgical eradication and radiotherapy are beneficial for early-stage HNSCC [6], 7]. However, because early-stage HNSCC has no evident symptoms, 70–80 % of patients with HNSCC are diagnosed with locally advanced or metastatic disease. Few treatment options are available for such patients, with immunotherapy and chemotherapy having unsatisfactory therapeutic effects [8], 9]. Therefore, there is an urgent need to identify novel drugs and therapeutic strategies to improve clinical outcomes in patients with HNSCC, a disease characterized by latent onset and aggressive behavior.

Cell death can be programmed through ferroptosis, a newly discovered method that differs from apoptosis, necrosis, and autophagy and depends on iron ions and lipid peroxidation [10]. The main mechanisms and signaling pathways of ferroptosis are complex and are closely related to cellular amino acid metabolism, lipid metabolism, and key metabolic enzymes [11]. Ferroptosis has emerged as a major research focus in recent years due to its significant role in the pathogenesis of various diseases, including neurodegenerative disorders (such as Parkinson’s, Alzheimer’s, and Huntington’s diseases), cardiovascular diseases, stroke, kidney injury, and cancer [12]. Because cancer can be treated by targeting amino acids, lipids, and iron metabolism-related signaling pathways, which are associated with ferroptosis, most studies have focused on investigating anticancer mechanisms targeting ferroptosis [13], 14]. In particular, treatment methods targeting ferroptosis are very effective for stubborn drug-resistant malignant tumors [15]. Additionally, some studies have shown that ferroptosis has gradually become a key strategy for the treatment of head and neck cancer [16], 17]. Multiple ferroptosis inducers or gene knockout-induced ferroptosis can increase the sensitivity of drug-resistant head and neck cancer cell lines and tongue squamous cell carcinoma cells to cisplatin, further confirming the important relationship between ferroptosis and tumor resistance through multiple mechanisms. Ferroptosis has also shown potential therapeutic effects against advanced local HNSCCs, such as squamous cell carcinoma of the mouth, tongue, and throat (OSCC and TSCC), and esophageal squamous cell carcinoma (ESCC) [18], [19], [20], [21]. Consequently, the development of predictive models for the diagnosis and treatment of HNSCC is crucial to advance research on ferroptosis in this malignancy.

Irofulven, a mushroom-derived hemiterpenoid derivative with alkylating properties, has been extensively investigated as a novel pro-apoptotic antitumor agent due to its ability to induce pre-mitotic apoptosis and interfere with the cell cycle in cancer cells [22]. In the early 2000s, a study proposed that the use of irofulven in combination with 5-fluorouracil and cisplatin in the treatment of colorectal and breast cancers significantly enhanced antitumor activity [23]. However, research on irofulven appeared to be stagnant in the last decade. Irofulven, which acts as a DNA-damaging agent, is prone to induce the failure of DNA repair mechanisms in cancers with DNA repair dysfunction, and various studies have attempted to avoid it because of its toxicity in animal experiments [22], 24]. However, recent studies have re-established the importance of irofulven, revealing new insights into its complex role in genome maintenance [23], 25]. Despite its established antitumor efficacy and potential for use in combination therapies, the impact of irofulven on ferroptosis in HNSCC, as well as its combinatorial effects with ferroptosis inducers in treating HNSCC, remains to be elucidated.

Our investigation focused on evaluating ferroptosis as a potential biomarker and treatment target for HNSCC. We constructed a ferroptosis-related prognostic model for HNSCC using bioinformatic approaches, and single-cell analysis revealed a significantly elevated risk score within the malignant tumor cell cluster. Additionally, integrated multi-omics profiling revealed significant correlations between the predictive model and three key factors: immune cell infiltration patterns, epigenetic modifications, and genomic alterations. Importantly, irofulven and RSL3 demonstrated significant synergistic antitumor effects against HNSCC in both *in vitro* and *in vivo* models.

## 2 Materials and methods

### 2.1 Data collection

The transcriptomic data of patients (GSE65858) were extracted from Affymetrix (GPL570 platform). The RMA algorithm was used to adjust the background. The study utilized TCGA-sourced molecular expression data and corresponding treatment outcomes from 547 HNSCC patients. Quantile normalization was used to normalize the samples. The TCGA cohort was used as the training set, and the GSE65858 dataset was used as the validation set. Additionally, data extracted from the GSE181919 dataset were used for single-cell RNA sequencing.

### 2.2 Hierarchical clustering

The ConsensusClusterPlus package in R was used for hierarchical clustering. HNSCC data from in order to observe the relationship between TCGA subgroups and ferroptosis, different subgroups were created. A maximum of 10 clusters were found, and a maximum of 3 clusters were found to be optimal. The heatmap was performed through ggplot2 and pheatmap packages in R.

### 2.3 Differentially expressed genes analysis

Data obtained via hierarchical clustering were normalized by limma package. Data were log2-transformed, and differentially expressed genes (DEGs) were identified. A log FC value of ≥1 and statistical significance was established at *p* < 0.05 for screening differentially regulated genes.

### 2.4 Gene set enrichment analysis

Gene set enrichment analysis (GSEA) was used to identify GO terms and KEGG signaling pathways associated with DEGs. Functional pathway enrichment analysis was performed using R package enrichplot and clusterProfiler, with statistical significance set at *p* < 0.05.

### 2.5 Construction of a risk score model via LASSO regression

The gene expression data and clinical information of patients in the TCGA-HNSCC cohort were used to establish a risk score model. First, univariate Cox proportional hazard regression analysis was performed to investigate the relationship between gene expression and overall survival (OS) using the survival package in R. A *p*-value of 0.05 was considered significant. To identify the most significant prognostic genes, LASSO regression analysis was performed using the glmnet package in R. The TCGA-HNSCC cohort was used as the training set, whereas the GSE65858 dataset was used as the validation set. A risk score was calculated using the following formula: risk score = ∑ni ExpiCoei (Exp = expression level; Coe = regression coefficient). Using the median risk score as the threshold, patients were stratified into low- and high-risk cohorts. Kaplan–Meier plots and the log-rank test was used to estimate and compare OS between the two risk groups. Statistical significance threshold was set at *p* < 0.05. Receiver operating characteristic (ROC) curve analysis and area under curve (AUC) computation were performed using the pROC library in R. Consequently, six risk score-related genes were found to be associated with the prognosis of HNSCC.

### 2.6 Single-cell RNA sequencing

ScRNA-seq was performed and the expression data extracted from GSE181919 were processed using the R software. The transcriptional heterogeneity of cells in HNSCC tissues was examined using the Seurat package in R. Data were integrated, pre-processed, and subjected to non-linear dimension reduction, and batch effects were reduced. The FindCluster function was used to cluster the cells, and the Findmarkers function was used to identify marker genes in the clusters. Automatic annotation of cells was performed using SingleR. Pseudotemporal trajectory analysis was performed using the Monocle3 package with default parameters. The Cellchat R package was used for cell-cell communication analysis.

### 2.7 Tumor-infiltrating immune cell analysis

The TCGA-HNSCC dataset was categorized into distinct risk subgroups (high vs. low), while the GSE127165 samples were classified as either tumor specimens or normal tissues. Immune cell composition was quantified across 22 subtypes using the Cibersort computational method, with statistical significance defined at *p* ≤ 0.05.

### 2.8 Analysis of sensitivity to chemotherapy

Drug sensitivity was analyzed using the CellMiner tool. Additionally, the association between prognostic gene signatures and therapeutic sensitivity was investigated.

### 2.9 Somatic mutation analysis

TCGA-derived mutation datasets for HNSCC cases were retrieved and subsequently processed. The R-based maftools module facilitated both data consolidation and visualization of genetic variants.

### 2.10 Molecular methylation analysis of DEGs

The study utilized publicly available HNSCC datasets from TCGA, encompassing RNA-seq data and methylation arrays. Data preprocessing and methylation analysis were executed using the ChAMP in R, whereas the DESeq2 package in R was used to examine the differential expression of mRNAs. After normalization and quality control of the data (Benjamini–Hochberg correction, *p* < 0.05), differential methylation sites (DMSs) were identified as cytosine-phosphate-guanine (CpG) sites with deltabeta values of >0.1, and differential methylation genes (DMGs) were identified as genes with differentially methylated sites (DMSs). DEGs were intersected with DMGs to identify genes affected by altered genes.

### 2.11 Cell culture

Human squamous carcinoma cell lines SCC25 and CAL27 were cultured in DMEM (Gibco, Grand Island, NY, USA) supplemented with 10 % fetal bovine serum in a 5 % CO_2_ incubator at 37 °C.

### 2.12 Colony formation assay

SCC25 and CAL27 cell lines were plated in 6-well culture dishes and maintained for two weeks. Cloning was completed when the number of clones reached >50 cells. After 30 min of fixation with 10 % paraformaldehyde, the cells were treated with crystal violet stain according to the manufacturer’s protocol (Hema 3 Stain Kit, Thermo Fisher).

### 2.13 CCK-8 assay

SCC25 and CAL27 cells were cultured in 96-well plates with 5,000 cells per well. The cells were cultured for 72 h and collected for subsequent analysis. Before 2 h of collection, the CCK-8 reagent was added to cells, and the cells were quantified on a VersaMax microplate reader at a wavelength of 450 nm (molecular devices).

### 2.14 Reactive oxygen species (ROS) assay

Lipid ROS levels were assessed using the fluorescent probe BODIPY 581/591C11 (Thermo Fisher Scientific, #D3861), which specifically binds oxidized lipids. Cells (1 × 10^5^/well) were plated in 12-well plates and maintained under standard culture conditions (37 °C, 5 % CO_2_). After incubation with 1.5 μM BODIPY probe for 30 min in darkness, cells were washed twice with chilled HBSS (Thermo Fisher Scientific, #14025076) and resuspended in fresh HBSS. Flow cytometry analysis (excitation/emission: 581/591 nm) was performed to quantify fluorescence shifts, reflecting lipid peroxidation intensity.

### 2.15 Iron assay

Intracellular Fe^2+^ levels were determined using a colorimetric Iron Assay Kit (ab83366, Abcam) following established protocols. Briefly, 5 × 10^6^ cells were plated in 10-cm dishes and treated with experimental compounds for 12 h. Cells were rinsed with PBS, homogenized in chilled assay buffer, and centrifuged (13,000 ×g, 10 min, 4 °C) to remove debris. The supernatant was mixed with an iron-reducing agent (30 min, RT) to convert Fe^3+^ to Fe^2+^, followed by incubation with the chromogenic probe (100 μL, 1 h, dark). Absorbance at 593 nm was recorded via microplate reader, with Fe^2+^ concentrations normalized to total protein content. This assay is validated in ferroptosis studies, where iron overload exacerbates lipid peroxidation and oxidative stress. For quality control, include blank controls and standard curves with each experimental batch.

### 2.16 Transmission electron microscopy

For ultrastructural examination of tumor tissues, samples were sectioned into 1 mm^3^ fragments and immediately immobilized in ice-cold 2.5 % glutaraldehyde/0.1 M PIPES buffer (pH 7.4) for 10 h at 4 °C. After PBS rinsing, secondary fixation was performed with 1 % osmium tetroxide (30 min, RT), followed by contrasting in 2 % uranyl acetate (1 h, RT). Dehydration was achieved through graded ethanol series (50 %, 70 %, 100 %), and tissues were embedded in epoxy resin (37 °C, 12–16 h). Ultrathin sections (70 nm) were prepared using a diamond knife and imaged under an HT-7700 TEM to visualize subcellular structures.

### 2.17 Mice

Male BALB/c nude mice (4 weeks old, 15 g body weight) were sourced from Guangdong Guangzhou Biotech (SPF-certified facility) and housed under controlled conditions (22–24 °C, 55 % humidity, 12 h light/dark cycle). All protocols adhered to ethical guidelines approved by the Institutional Animal Care and Use Committee (IACUC) of Sun Yat-sen University Cancer Center (approval #L025501202506004). To establish HNSCC xenografts, 5 × 10^6^ CAL27 cells suspended in 100 μL PBS were subcutaneously injected into the right flank of each mouse. Tumor dimensions were measured biweekly using calipers, and volumes were calculated as (length × width^2^)/2. When tumors reached 50 mm^3^, mice were randomized into four cohorts (*n* = 5/group): (1) vehicle control DMSO group, (2) Irofulven group, (3) RSL3 group, and (4) Irofulven + RSL3 combination group. Treatments were administered via daily oral gavage for 28 days. Terminal euthanasia was performed with intraperitoneal pentobarbital (150 mg/kg), followed by tumor excision, weight measurement, and downstream histopathological or molecular analyses.

**Ethical approval:** The research related to animal use has been complied with all the relevant national regulations and institutional policies for the care and use of animals, and has been approved by the Experimental Animal Ethics Committee: Sun Yat-sen University (Approved protocol number: L025501202506004).

### 2.18 IHC

Tumor specimens were immobilized in 10 % neutral buffered formalin (Solaibio #G2160) for 24 h at 4 °C and processed into paraffin-embedded blocks (Solaibio #YA0012) following standardized histopathological workflows. Serial sections (4 μm) were mounted on slides, dewaxed in xylene, and rehydrated through graded ethanol. Antigen retrieval was performed via heat-induced epitope retrieval (HIER) in citrate buffer (pH 6.0, 95 °C, 20 min) to restore Ki67 epitope accessibility. Endogenous peroxidase activity was quenched with 3 % H_2_O_2_ (10 min, RT), followed by blocking in 5 % BSA. Sections were incubated with anti-Ki67 primary antibody (Cell Signaling Technology #9027, 1:800 dilution) overnight at 4 °C. After PBS washes, HRP-conjugated secondary antibody (Cell Signaling Technology #8114S, 1:1 dilution) was applied for 60 min at 25 °C. Digital pathology systems quantified Ki67 positivity (%) across five high-power fields (HPFs) per sample.

### 2.19 Artificial intelligence tools

No artificial intelligence (AI) generative tools – including but not limited to large language models such as ChatGPT, Google Bard, Claude, or similar systems – were utilized for content generation, text rewriting, data analysis, image creation, experimental design, or any other aspect of the research and manuscript preparation process. The authors are solely responsible for the conception, execution, data interpretation, manuscript composition, critical revision, and final editing of this work.

### 2.20 Statistical analysis

Statistical analyses were conducted to assess overall survival (OS) differences between the two risk groups. In the training cohort, Kaplan–Meier curves were generated and compared using the log-rank test. For the validation cohort, OS differences were evaluated with the Renyi test. Hazard ratios and corresponding *p*-values were derived from Cox proportional hazards regression. Associations between variables were examined via Spearman’s rank correlation. All statistical computations were performed using GraphPad Prism 8.0. Continuous data are expressed as mean ± standard deviation from three independent replicates. Comparisons of two groups were performed using an unpaired two-tailed Student’s *t*-test. For comparisons among more than two groups, one-way ANOVA with Tukey’s multiple comparisons test was applied. When two independent variables were present across multiple groups, two-way ANOVA followed by Bonferroni’s post hoc test was used. Longitudinal tumor volume measurements across different treatment arms were analyzed by two-way repeated-measures ANOVA with Bonferroni correction. Immune cell infiltration estimates, obtained from CIBERSORT analysis, were compared using the Wilcoxon rank-sum test. A *p*-value < 0.05 was considered statistically significant.

## 3 Results

### 3.1 Consensus clustering of ferroptosis-related genes in patients with HNSCC

**Figure 1:**
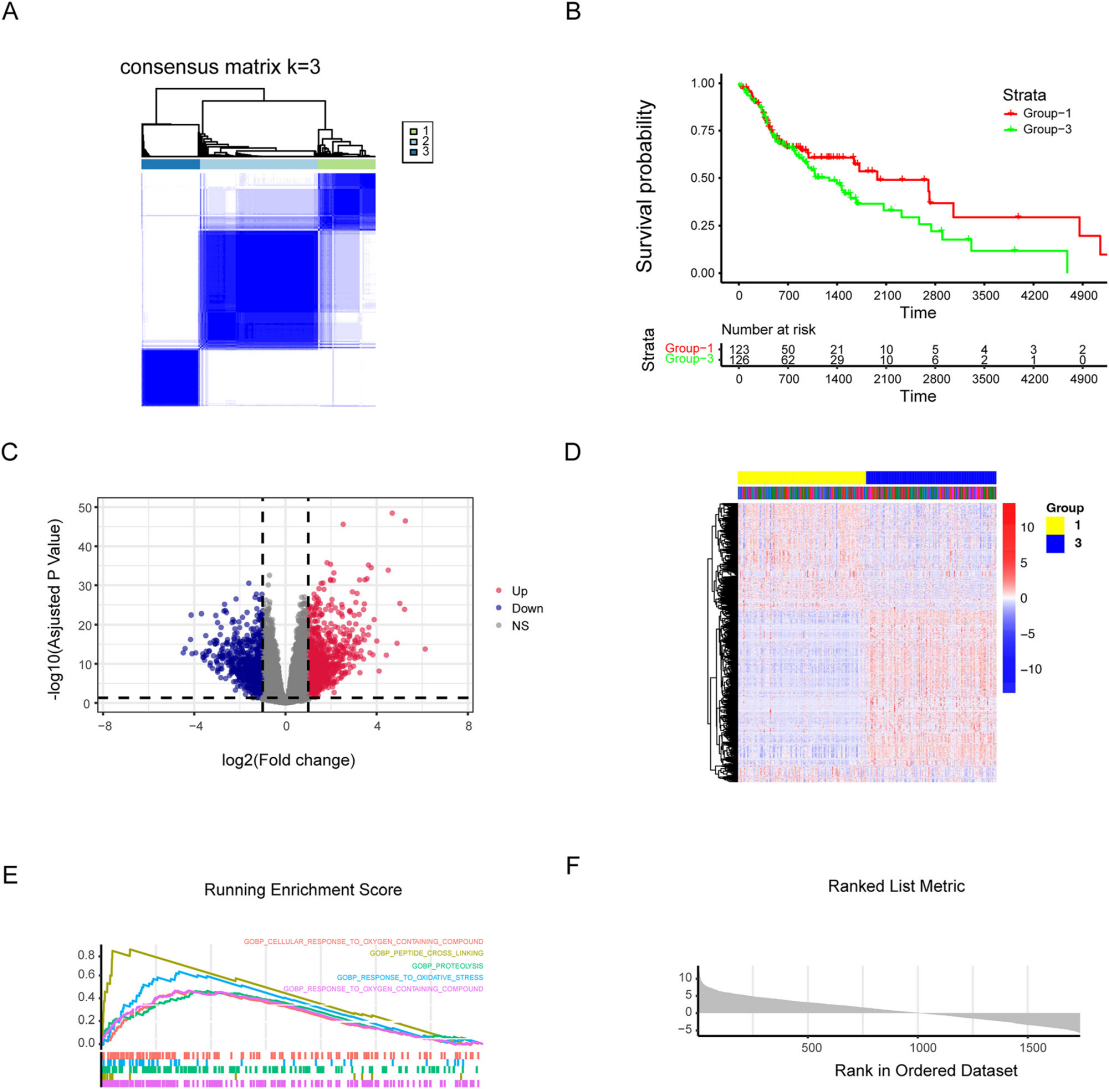
The consensus cluster analysis of ferroptosis-related genes. (A) The consensus matrix of all samples when *k* = 3; (B) Kaplan–Meier curves of progression-free survival probability between two clusters. (C–D). Volcano plot (C) and heatmap (D) of ferroptosis-related DEGs between group 1 and group 3. The blue represents low-expressed DEGs and the red represents over-expressed DEGs. (E) The enrichment scores of the top five ferroptosis-related gene sets were enriched through GSEA. (F) The ranked list metric of the top five ferroptosis-related gene sets were enriched through GSEA. DEGs, differentially expressed genes; GSEA, gene set enrichment analysis.

The initiation and progression of various cancers are closely linked to ferroptosis pathways. To investigate the role of ferroptosis, we performed consensus clustering analysis (*k* = 3) on gene expression profiles from 504 HNSCC patients in the TCGA-HNSCC cohort, which stratified the samples into distinct subgroups (Figure 1A and Supplementary Figure S1A–B). Kaplan–Meier analysis revealed that survival was better in group 1 than in group 3 (Figure 1B). DEGs were identified based on these results. A total of 986 upregulated genes and 752 downregulated genes were identified using the limma package (Figure 1C and D and Supplementary Figure S1C). Functional annotation via GSEA revealed that the differentially expressed genes were primarily enriched in pathways related to oxygen response, oxidative stress, peptide cross-linking, and proteolysis (Figure 1E and F and Supplementary Figure S1D).

### 3.2 LASSO regression analysis for patients with HNSCC

**Figure 2:**
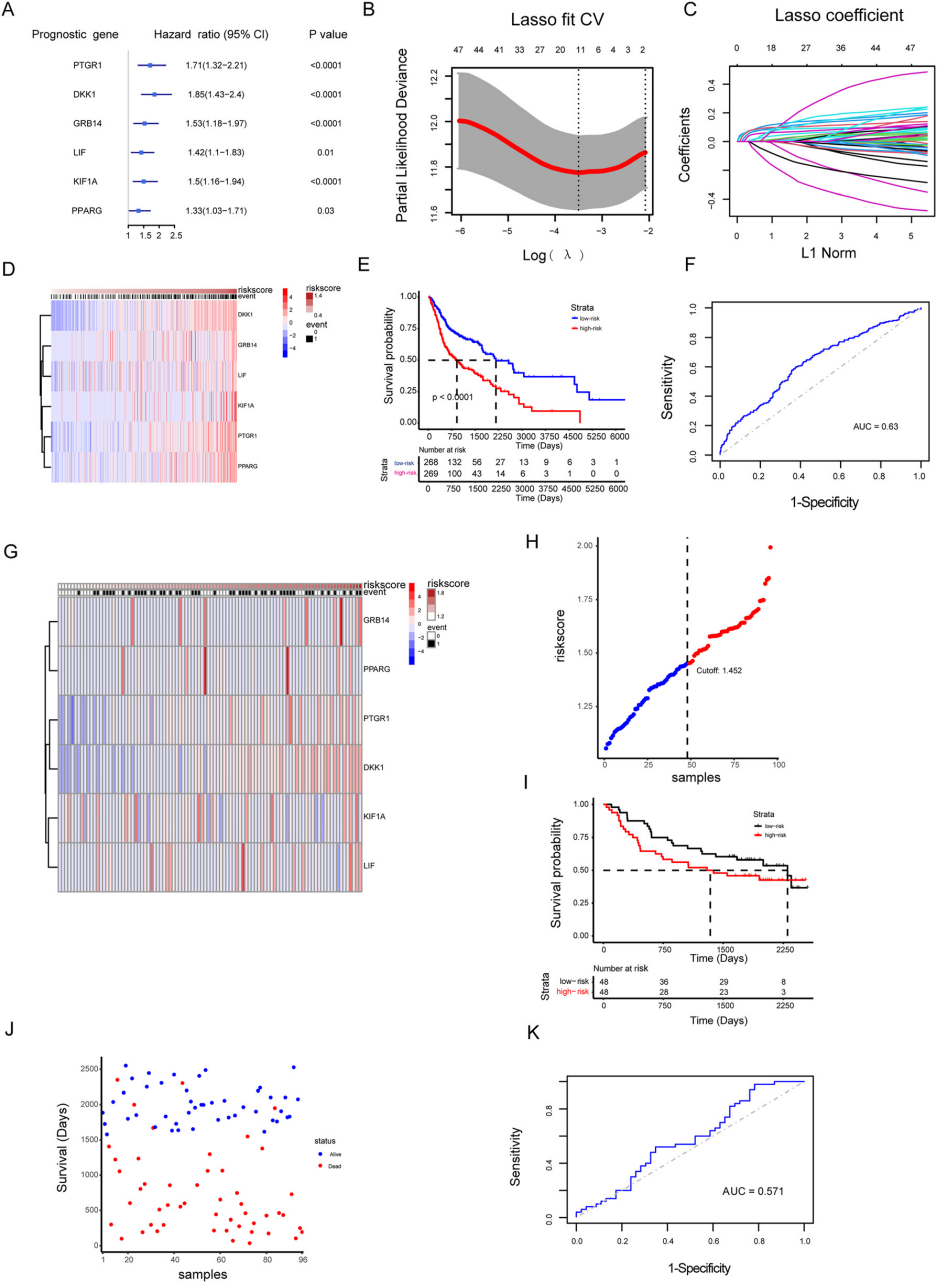
LASSO regression and risk score calculation of TCGA-HNSCC training set. (A) The forest plot of differentially expressed genes of risk score in the TCGA training set. (B) LASSO deviance profile of the 48 intersection genes. (C) LASSO regression coefficient profile of the 48 intersection genes. (D) Risk score in the TCGA training set, patient survival, and expression of 6 DEGs in the TCGA training set. (E) Risk score and survival probabilities in the TCGA training set. (F) Receiver operating characteristic curve (ROC) of risk score in the TCGA training set. (G) Risk score in the GEO test set, patient survival, and expression of 6 DEGs in the GEO test set. (H) Distribution of risk score between low and high-risk groups in the GEO test set. (I) Risk score and survival probabilities in the GEO test set. (J) The survival status of patients in the GEO test set. (K) ROC of risk score in the GEO test set.

Univariate Cox regression identified 1,004 oncogenic factors significantly correlated with HNSCC progression (hazard ratio >1, *p*-value < 0.05). These genes were intersected with the upregulated genes in TCGA-HNSCC cohort, and a total of 537 candidate genes were identified (Figure 2A). To minimize the impact of false-positive findings, LASSO regression analysis was conducted using TCGA-HNSCC patient data for model training, followed by cross-validation with the independent GEO dataset GSE65858.

In the training set, six genes were identified (Figure 2A), and the risk score was calculated as follows: Risk score = 0.0456 × expression (growth factor receptor bound protein 14, GRB14) + 0.0269 × expression (kinesin family member 1A, KIF1A) + 0.0643 × expression (peroxisome proliferator activated receptor gamma, PPARG) + 0.0666 × expression (PTGR1) + 0.0691 × expression (dickkopf wnt signaling pathway inhibitor 1, DKK1) + 0.0012 × expression (leukemia inhibitory factor, LIF) for the 6 DEGs in the TCGA training set (Figure 2D). LASSO deviance profile and LASSO regression coefficient profile of the 48 intersecting genes were shown in Figure 2B and C. Median values were calculated based on the critical value of high-risk and low-risk groups (Supplementary Figure S2A–C). Patients stratified into high- and low-risk categories exhibited markedly distinct survival outcomes in the training cohort (Figure 2E). The AUC value was 0.63 over the past year (Figure 2F). Consistent findings were replicated in the independent validation cohort. (Figure 2G–K).

### 3.3 Biological functions of genes related to risk scores examined via single-cell sequencing

**Figure 3:**
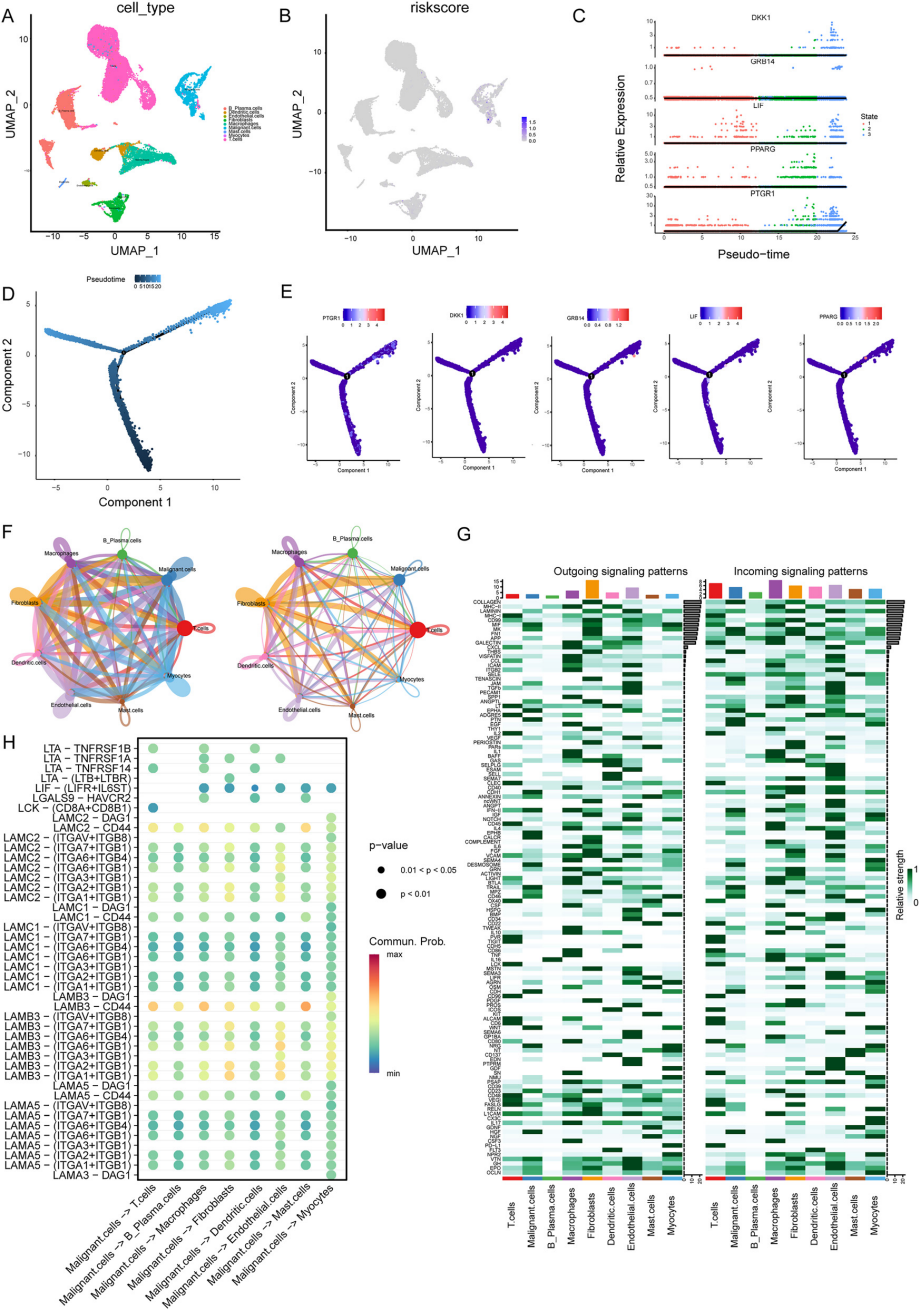
scRNA-seq analysis of risk score and risk-score genes in HNSCC. (A) The HNSCC cells were categorized into 9 clusters. (B) Scatter plots of the risk score expression distribution in 9 clusters. (C) Relative expression value of five risk score-related genes in 3 cell states based on pseudotime. 1–3 represents the development period of tumors, which was divided into three stages, from 1 to 3. (D) The pseudotime trajectories of macrophages containing three main branches. (E) The trajectory of five risk score-related genes in macrophages containing three main branches. (F) The interaction weights/strength (left) and number of interactions between different cell groups. (G) The outgoing signaling patterns and incoming signaling patterns between different cell groups. (H) The heatmap of ligand-receptor expression between cell groups.

Furthermore, the risk scores and five risk score-related genes were examined via single-cell sequencing. Uniform manifold approximation and projection (UMAP) analysis revealed a dense distribution of risk scores within malignant cells (Figure 3A and B). To obtain a dynamic view of malignant evolution, we reconstructed a single-cell trajectory of the malignant epithelial compartment and rooted it in the epithelial-like substate displaying the lowest risk scores (state 1), with progression toward more aggressive terminal states (states 2–3) (Figure 3C–E). This trajectory is consistent with a transition from an epithelial/low-malignancy program to partially EMT/stress-responsive (state 2) and mesenchymal/invasive, microenvironment-interactive states (state 3). Mapping the five risk score-related genes onto the trajectory revealed stage-specific induction patterns. Consistent with these dynamics, the aggregate risk score increased monotonically from state 1 to states 2–3 (Figure 3C), and cells in terminal states were enriched for the top 70 cluster-defining genes associated with invasive and microenvironment-interactive programs (Supplementary Figure S3A). And the risk score-related genes were highly enriched in malignant cells, macrophages, and fibroblasts (Supplementary Figure S3B). Furthermore, we performed cell-cell communication analysis, which revealed prominent interactions between tumor cells and macrophages, endothelial cells, and fibroblasts (Figure 3F and G). Notably, the LASSO-identified gene LIF exhibited significant communication links between tumor cells and macrophages, as well as between tumor cells and endothelial cells (Figure 3H). These findings suggest that LIF may play a regulatory role in mediating crosstalk between tumor cells and both macrophages and endothelial cells.

### 3.4 Tumor microenvironment analysis in HNSCC

**Figure 4:**
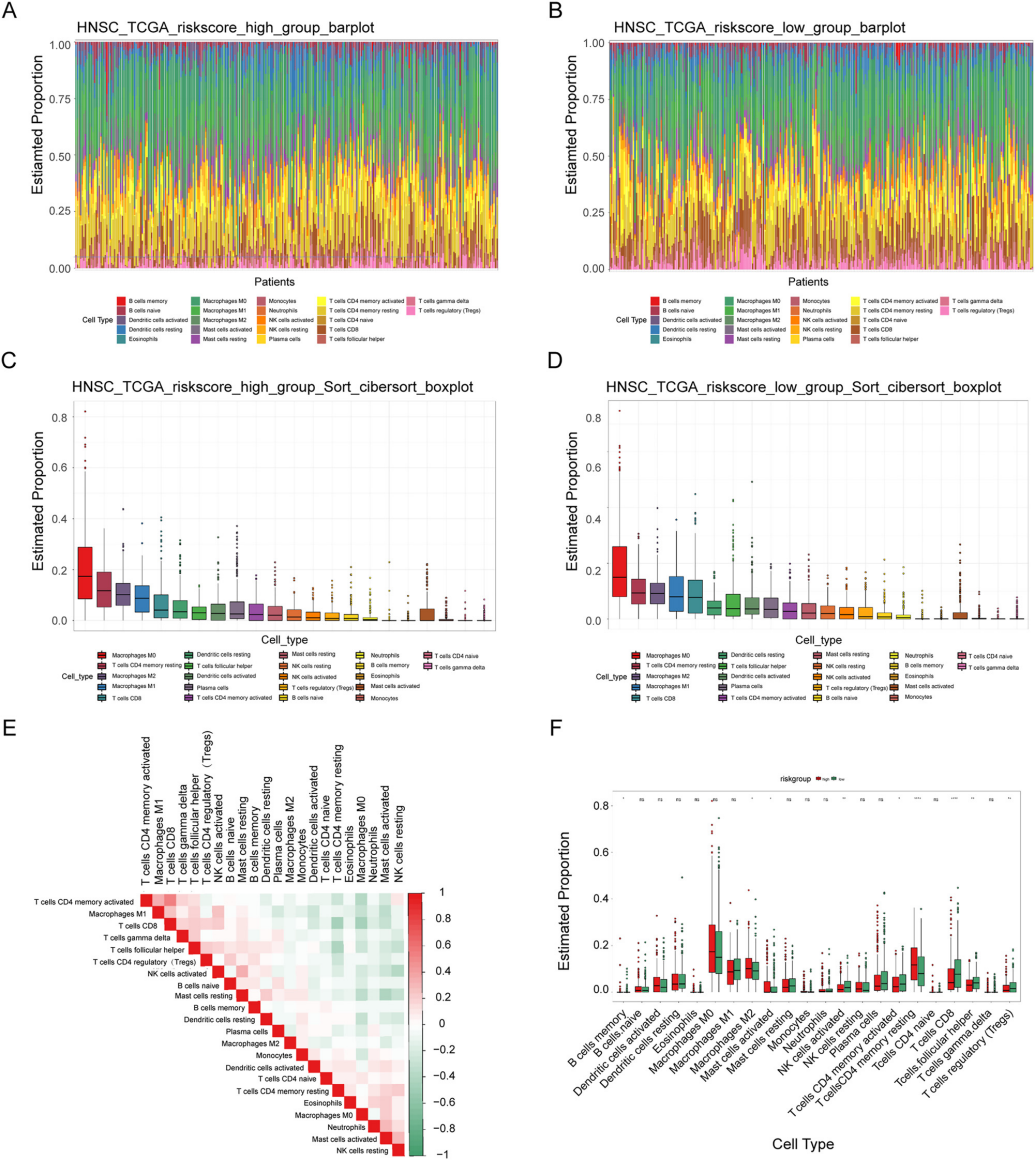
The immune infiltration of 22 immune cell types in high- and low-rick score HNSCC cohort. (A) The mean proportion of 22 immune cell types in the high-risk score HNSCC patient tissues. (B) The mean proportion of 22 immune cell types in the low-risk score HNSCC patient tissues. (C) The boxplot of the enriched proportion of 22 immune cell types in the high-risk score HNSCC patient tissues. (D) The boxplot of enriched proportion of 22 immune cell types in the low-risk score HNSCC patient tissues. (E) Correlation matrix of all 22 immune cell proportions. (F) The differentiation of 22 immune cell types between high and low-risk score HNSCC cohorts. Immune cell infiltration estimates were compared using the Wilcoxon rank-sum test (* *p* < 0.05, ** *p* < 0.01, *** *p* < 0.001, **** *p* < 0.0001).

The proportion of immune cells in the tumor microenvironment was analyzed to determine whether the prognostic model effectively reflected the status of the tumor immune microenvironment in HNSCC. The proportion of different immune cell types in the high- and low-risk groups is shown in Figure 4A–D. A correlation was observed among all cell types (Figure 4E). A similar analysis was performed in the normal control and HNSCC groups (Supplementary Figure S4A–E). As shown in Figure 4F, the infiltration levels of activated dendritic cells, M1 macrophages, activated mast cells, monocytes, neutrophils, and CD8+ T cells were different between the high- and low-risk groups. Notably, the infiltration levels of activated memory CD4+ T cells and CD8+ T cells differed markedly between normal and tumor tissues (Supplementary Figure S4F), indicating significant remodeling of the immune microenvironment in HNSCC, which our prognostic model can partially predict.

### 3.5 DMSs analysis and somatic mutation analysis in HNSCC

**Figure 5:**
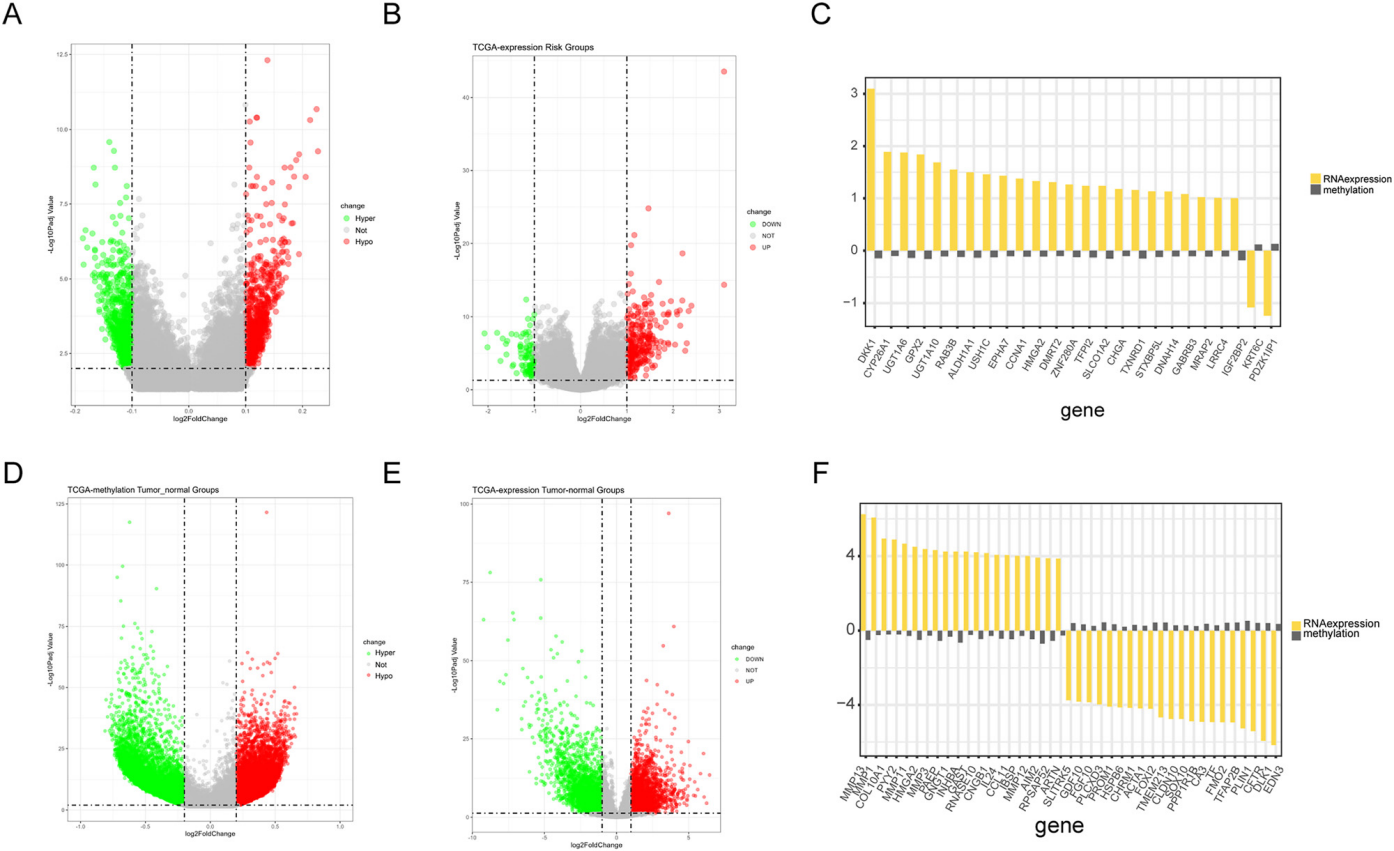
Identification of DEGs correlated with DMS in HNSCC patients. (A) Volcano plot of DMSs in high- and low-risk groups of HNSCC patients. (B) Volcano plot of DEGs in high- and low-risk groups of HNSCC patients. (C) The 25 differentially expressed and methylated genes with the negative correlation in the high-risk group compared with the low-risk group of HNSCC. (D) Volcano plot of DMSs between normal control and HNSCC patients. (E) Volcano plot of DMSs between normal control and HNSCC patients. (F) The 25 pairs of DEGs and DMS with negative correlations in normal control and HNSCC patients.

DMSs between the high- and low-risk groups were identified with ChAMP and DEGs with DESeq2, yielding 1,741 DMSs (937 hypermethylated, 804 hypomethylated) and 391 DEGs (298 upregulated, 93 downregulated; Figure 5A and B). Of the DEGs, 25 DEGs were associated with DMSs: 23 upregulated genes with hypomethylation and two downregulated genes with hypermethylation (Figure 5C). In the comparison between normal controls and cancers, we identified 67,542 DMSs (14,240 hypermethylated, 53,302 hypomethylated) and 7,325 DEGs (3,597 upregulated, 2,728 downregulated; Figure 5D and E), of which 41 DEGs were associated with DMSs (20 upregulated with hypomethylation and 21 downregulated with hypermethylation; Figure 5F).

The impact of gene mutations on the prognostic model was examined. The maftools package in R was used to analyze and visualize gene mutation data in TCGA-HNSCC cohort. The mutation frequency of tumor protein 53 (TP53) and titin (TTN) was highest in all patients with HNSCC (Supplementary Figure S5A). Based on prognostic risk scores, patients were stratified into low- and high-risk groups; TP53, TTN, and FAT atypical cadherin 1 (FAT1) mutations were most frequent in the low-risk group, whereas TP53, TTN, and cyclin dependent kinase inhibitor 2A (CDKN2A) mutations predominated in the high-risk group (Supplementary Figure S5B-C).

### 3.6 Exploration of drug sensitivity in the prognostic model

**Figure 6:**
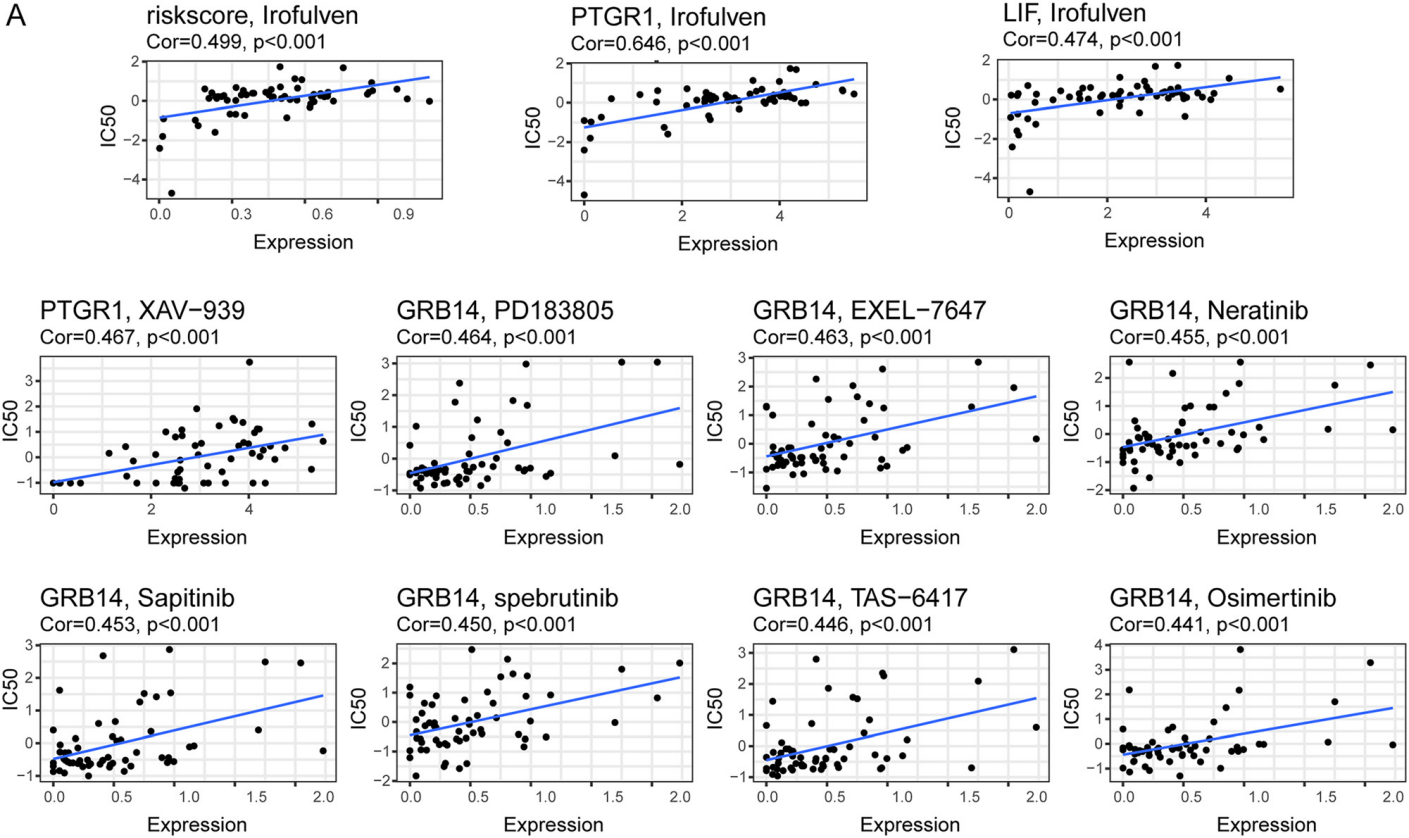
Drug sensitivity analysis of prognostic genes based on the CellMiner database. Drug sensitivity analysis through the correlation analysis of drug *z*-score with risk score and prognostic gene expression.

To nominate therapeutic candidates, we intersected model-derived prognostic genes with drug response profiles in the CellMiner database. Our analysis revealed 16 drug candidates that were strongly linked to outcome-related genes (*p* < 0.05, *r* > 0.3), with results visualized in Figure 6. PTGR1 was found to be most sensitive to irofulven (cor = 0.646).

### 3.7 Silencing PTGR1 enhances HNSCC ferroptosis

**Figure 7:**
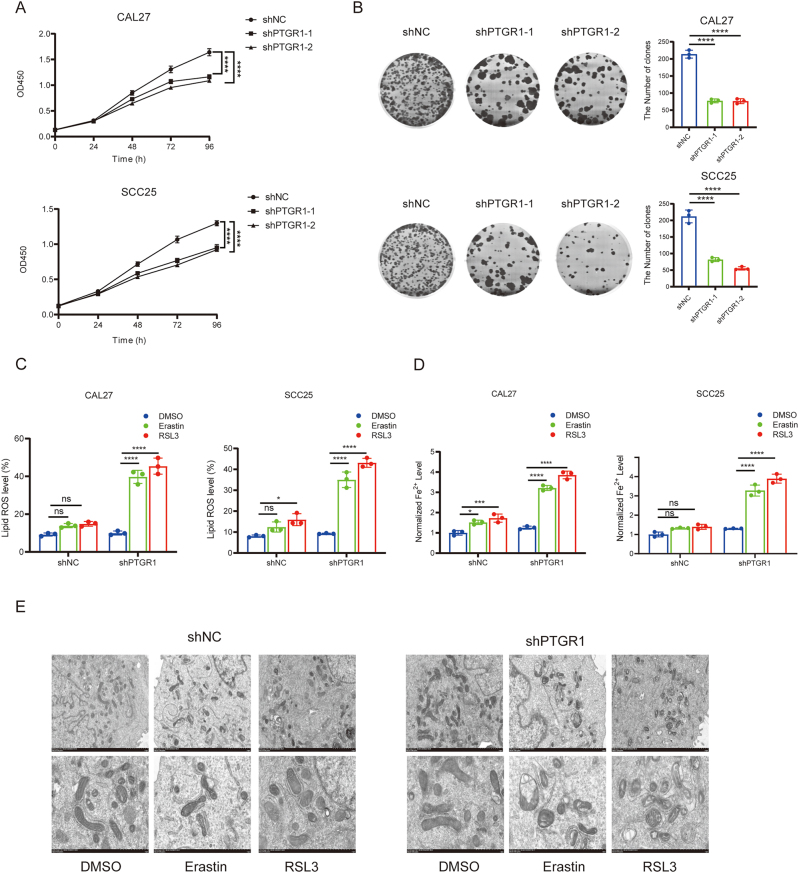
Silencing PTGR1 enhances HNSCC ferroptosis. (A) The CCK-8 assays were performed in shNC and shPTGR1 CAL27 and SCC25 cell lines. (B) The colony formation assays were performed in shNC and shPTGR1 CAL27 and SCC25 cell lines. (C) Lipid ROS level of shNC and shPTGR1 CAL27 and SCC25 cell while exposure to low-dose Erastin (2 μM) or RSL3 (1 μM) for 24 h. (D) Fe^2+^ level of shNC and shPTGR1 CAL27 and SCC25 cell while exposure to low-dose Erastin (2 μM) or RSL3 (1 μM) for 24 h. (E) Transmission electron microscopy photographed while exposure to low-dose Erastin (2 μM) or RSL3 (1 μM) for 24 h in shNC or shPTGR1 CAL27 cells. Values are expressed as the means ± SD from three experiments, and the asterisk indicates the statistical significance compared to the controls (* *p* < 0.05, ** *p* < 0.01, *** *p* < 0.001, **** *p* < 0.0001).

To further investigate the role of PTGR1 in HNSCC, we examined its impact on HNSCC cells by knocking down PTGR1 and conducting CCK-8 and colony formation assays. PTGR1 knockdown significantly suppressed HNSCC cell proliferation, as evidenced by CCK-8 and colony formation assays (Figure 7A and B). Additionally, PTGR1 is one of the genes in the ferroptosis-related prognostic model for HNSCC, therefore we further explored its influence on ferroptosis levels in HNSCC. Using flow cytometry to measure ROS levels, we found that low concentrations of Erastin and RSL3 significantly increased ROS levels in the PTGR1 knockdown group, whereas no significant changes were observed in the control group (Figure 7C). Concurrently, we also measured Fe^2+^ levels in HNSCC cells, and the results were consistent with the aforementioned findings (Figure 7D). Furthermore, we employed transmission electron microscopy to assess mitochondrial morphology in HNSCC cells following PTGR1 knockdown. The results revealed that treatment with low concentrations of Erastin and RSL3 led to a marked increase in mitochondrial shrinkage in the PTGR1 knockdown group, while no significant changes were observed in control group (Figure 7E). Collectively, these findings demonstrate that PTGR1 knockdown sensitizes HNSCC cells to ferroptosis.

### 3.8 Combination of irofulven and RSL3 for the treatment of HNSCC *in vitro*

**Figure 8:**
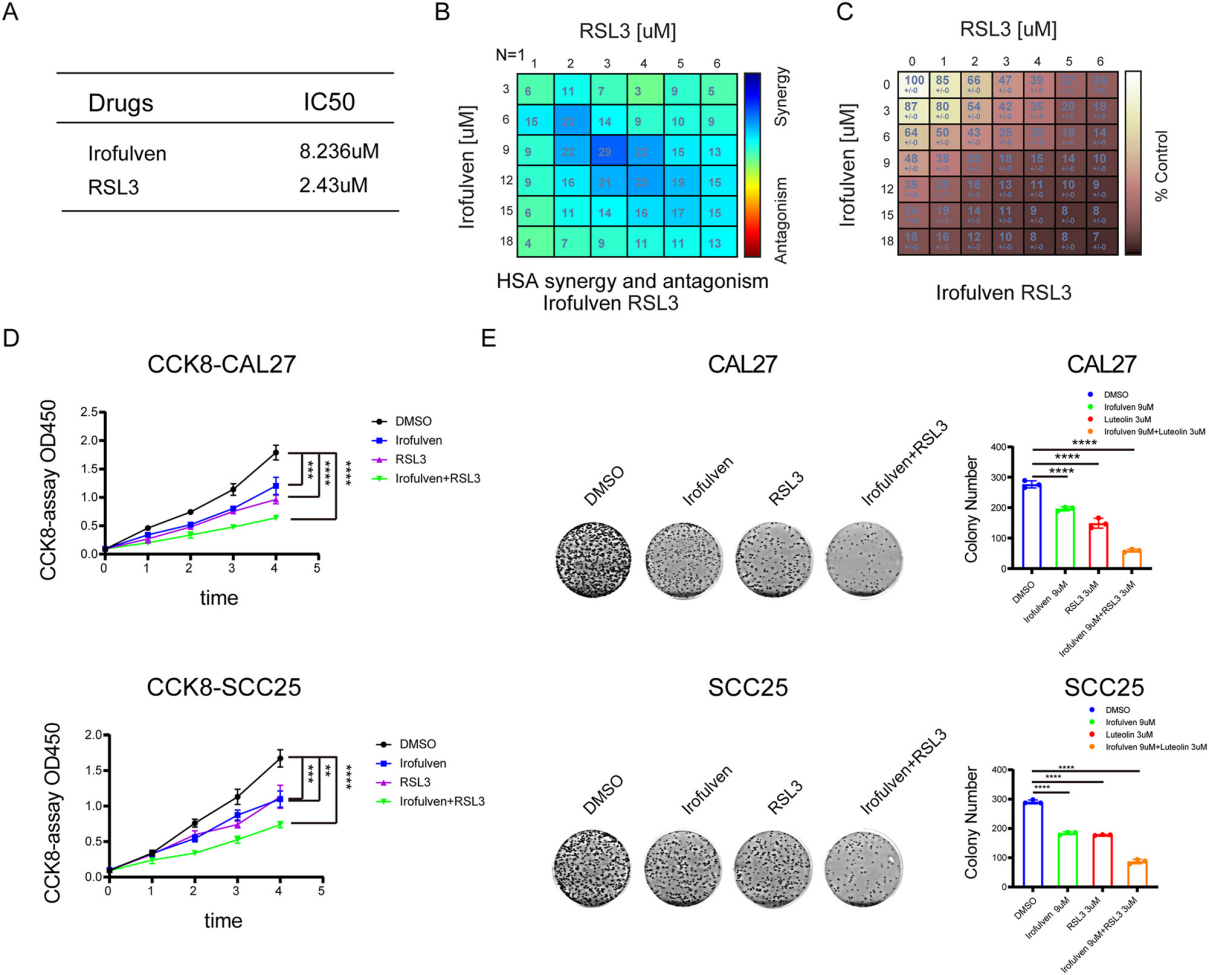
The combination of Irofulven and RSL3 for the treatment of HNSCC *in vitro*. (A) IC50 of Irofulven and RSL3. (B–C) The Cell Viability Assay monitored proliferation of the indicated cell lines. The two cell lines were treated with increasing doses of Irofulven in combination with increasing doses of RSL3. (D) CCK8 proliferation assay of the indicated cell lines with the treatment of Irofulven and RSL3. (E) Irofulven and RSL3 synergistically inhibited cell clone formation. The indicated cell lines were exposed to Irofulven and RSL3, and the clone numbers were counted. Values are expressed as the means ± SD from three experiments, and the asterisk indicates the statistical significance compared to the controls (* *p* < 0.05, ** *p* < 0.01, *** *p* < 0.001, **** *p* < 0.0001).

CAL27 and SCC25 cells, derived from squamous cell carcinoma tissues, serve as established models for HNSCC cellular behavior. The IC50 values of these two compounds were evaluated via CCK8 assay (Figure 8A). Based on the IC50 values, irofulven and RSL3 were selected for subsequent experiments. Both cell lines were treated with the combination of irofulven and RSL3. Compared with the use of either drug alone, the combination of the two drugs reduced cell viability more effectively (Figure 8B and C). Similar results were observed in CCK8 and colony formation assays (Figure 8D and E). Therefore, the combination of irofulven and RSL3 can effectively inhibit the growth of tumor cells.

### 3.9 Combination of irofulven and RSL3 for the treatment of HNSCC *in vivo*

**Figure 9:**
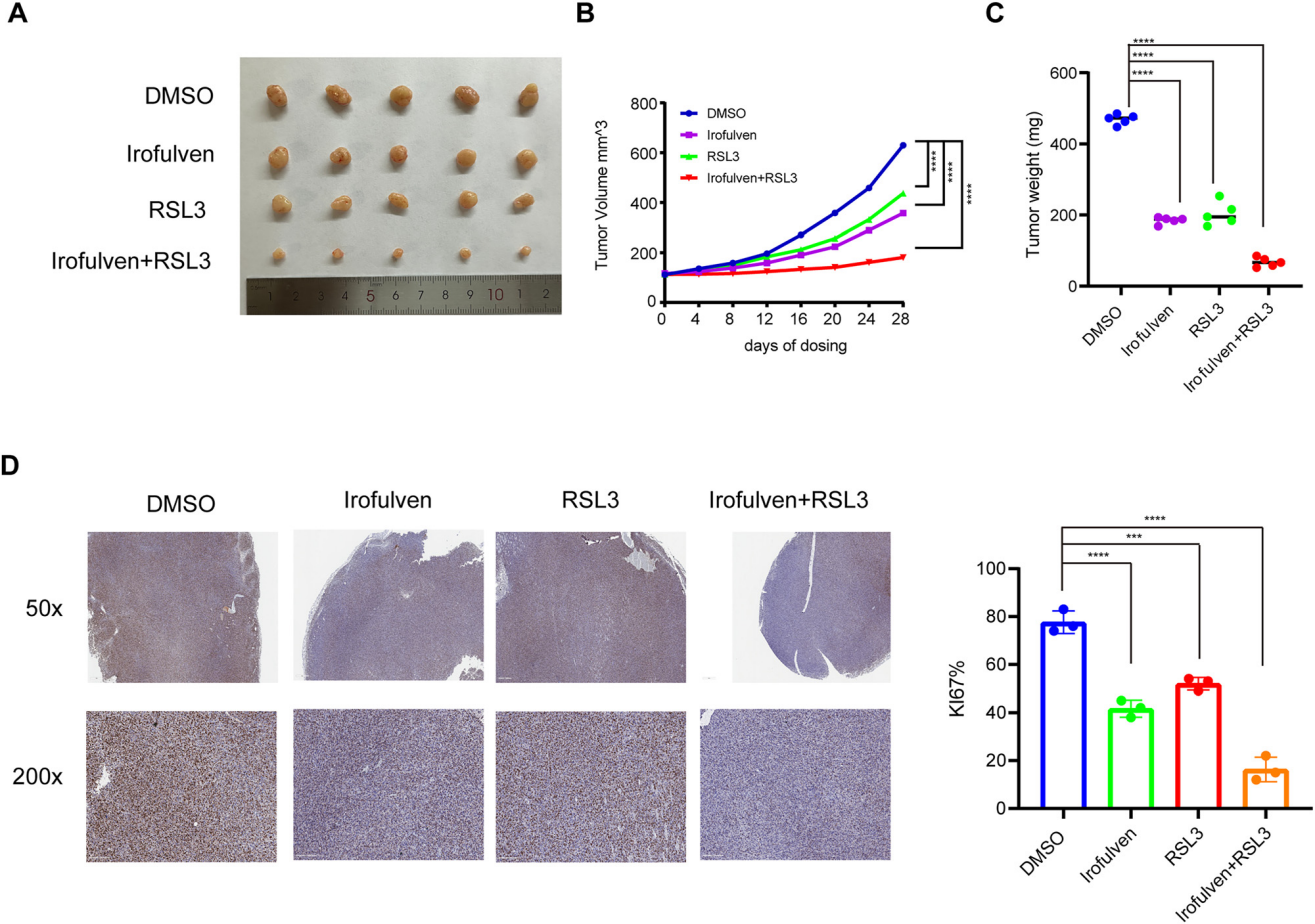
The combination of Irofulven and RSL3 for the treatment of HNSCC *in vivo*. (A) The mice were treated with DMSO, Irofulven, RSL3, Irofulven and RSL3, respectively, for 28 days. Four groups of mouse tumor photos. (B) The change trend of tumor volume in mice treated with DMSO, Irofulven, RSL3, and Irofulven and RSL3, respectively. (C) The tumor weights across the four groups. (D) The expression of the tumor species Ki67 in the mice after treatment with the four drugs was detected by immunohistochemistry. Values are expressed as the means ± SD from three experiments, and the asterisk indicates the statistical significance compared to the controls (* *p* < 0.05, ** *p* < 0.01, *** *p* < 0.001, **** *p* < 0.0001).

To further validate the therapeutic efficacy of the combined treatment with irofulven and RSL3 *in vivo*, we established a xenograft mouse model. Mice were randomly assigned to four groups (*n* = 6 per group): vehicle (DMSO), irofulven monotherapy, RSL3 monotherapy, or irofulven plus RSL3 combination therapy. The mice were treated with the respective drugs for a total of 28 days, and tumor size was measured every 4 days. The experimental results demonstrated that the tumor volume and weight in the combination treatment group were significantly lower than those in the single-treatment groups and the control group (Figure 9A–C). Furthermore, we assessed the expression level of Ki-67 in the tumor tissues, which also revealed a significant reduction in Ki-67 levels in the combination treatment group compared to the single-treatment groups and the control group (Figure 9D). These findings collectively indicate that the combined use of irofulven and RSL3 exerts a synergistic therapeutic effect on HNSCC *in vivo*.

## 4 Discussion

HNSCC is the most common and aggressive form of head and neck cancer. Therefore, it is necessary to find drugs and develop therapeutic methods that can improve the treatment of latent and invasive HNSCCs. Ferroptosis is a unique form of regulated cell death driven by iron-dependent lipid peroxidation, distinct mechanistically from apoptosis, necrosis, and autophagy. Activating ferroptosis for treating cancer is a major research focus, especially for intractable drug-resistant malignant tumors, and treatment methods based on ferroptosis have been demonstrated to be effective and potentially curative. Some studies have shown that ferroptosis has gradually become a potential strategy for the treatment of HNSCC. However, ferroptosis targets and ferroptosis-based methods for the diagnosis and treatment of HNSCC remain unclear. Establishing a predictive model for diagnosis and treatment is also considered a cornerstone of advancing the research on ferroptosis in HNSCC. In this study, we constructed a prognostic model based on ferroptosis-related genes via LASSO. Based on the results of single-cell sequencing, we examined the types of HNSCC and the related risk factors. Multi-omic analysis showed that the prognosis was correlated with immune infiltration, methylation, and DNA mutation. Through CellMiner database, we found irofulven could target the risk score-related gene PTGR1. Furthermore, silencing PTGR1 increases the ferroptosis levels in HNSCC. Notably, the combination of irofulven and RSL3 could synergistically treat HNSCC *in vitro* and *in vivo*. This study introduces a novel prognostic model for HNSCC, identifying key molecular targets and synergistic therapeutic strategies.

Utilizing expression profiles of 259 ferroptosis-associated genes in HNSCC samples, we conducted clustering and Cox regression analyses, followed by LASSO-selected gene incorporation into a prognostic scoring system. Most of the six genes in the risk model, PTGR1, DKK11, GRB14, LIF, KIF1A, and PPARG, are associated with various cancers. However, the role of some genes in HNSCC remains unclear. PTGR1 encodes a rate-limiting enzyme involved in the inactivation of the chemokine leukotriene B4 [26]. Beyond its established functions, this factor significantly contributes to tumor initiation and progression, positioning it as a promising therapeutic focus in oncology [27]. For example, PTGR1 knockdown directly affects the cell cycle and proliferation of prostate cancer cells [28], and PTGR1 overexpression is associated with a poor prognosis in various tumor types, including HNSCC [29]. DKK1 (an inhibitor of the WNT signaling pathway) encodes a protein that mediates protein-protein interactions and binds to the LRP6 co-receptor to inhibit catenin signaling. DKK1 promotes cancer cell growth, proliferation, and invasion, with elevated expression observed across multiple human malignancies [30]. GRB14 encodes a coupling protein that regulates cell surface receptor kinases and specific signaling pathways, which interact with insulin receptors and insulin-related growth factor receptors [31]. Studies have shown that GRB14 expression is positively correlated with the aggressive behavior of primary thyroid cancer [32]. LIF (an interleukin six family cytokine) is a leukemia- and myeloid leukemia-associated gene. In 2019, LIF was identified as a key paracrine factor found in activated pancreatic stellate cells that acts on cancer cells [33], 34]. KIF1A encodes a motor protein that transports membrane organelles along axonal microtubules as an anterograde motor protein. Some studies have suggested that KIF1A can be used in combination with other genes as a tumor marker for detecting HNSCC [35]. The protein encoded by PPARG regulates adipocyte differentiation and regulates energy metabolism, cell cycle, cell differentiation, and apoptosis [36]. PPARγ agonists have significant inhibitory effects on tumor cell proliferation *in vitro*; however, the anticancer efficacy of PPARγ agonists is not evident *in vivo*. Moreover, PPARγ agonists have not shown ideal therapeutic effects in clinical trials [37]. However, numerous studies have validated that when PPARγ ligands are used in combination with anti-cancer therapies, including chemotherapy, hormone therapy, targeted therapy, and immunotherapy, they often exert excellent anticancer effects [38]. In recent years, PPARγ activators have been used as chemotherapeutic targets for HNSCC because they can induce adipocyte differentiation of preneoplastic cell lines and reduce secondary cancer cell proliferation [39]. Therefore, the abovementioned six genes can be used for the diagnosis and treatment of HNSCC. The practicality and credibility of the prognostic model constructed in this study were assessed and verified in the training and validation sets, respectively, and the survival of patients with HNSCC was analyzed by plotting KM curves and calculating AUC values. Model-predicted prognoses aligned closely with observed patient outcomes.

In the era of advanced sequencing technologies, scRNA-seq has emerged as a widely adopted method for detecting transcriptional changes at the individual cell level [40]. scRNA-seq, which allows comprehensive classification of cell types, has considerably improved our understanding of biological systems [41]. In this study, we classified HNSCC samples via single-cell sequencing and divided them into five categories: A, B, C, D, and E. Among these five categories, category C (tumor body) had the highest risk score, indicating that risk is the feasibility of scoring to judge the process of tumorigenesis. Pseudotime trajectory analysis further showed that risk scores and model gene expression progressively increased along HNSCC developmental stages, confirming the model’s utility in predicting tumor initiation and progression. Therefore, scRNA-seq verified the ferroptosis-related prognostic model.

The tumor microenvironment (TME), as a complex ecosystem that sustains neoplastic proliferation, profoundly impacts oncogenesis, clinical prognosis, and the efficacy of immunotherapies [42]. TME is an influential factor affecting the coordination of tumor progression, immune escape, and resistance to immunotherapy in HNSCC [42]. Analyzing tumor-infiltrating immune cells using RNA sequencing data can provide an accurate assessment of the composition of immune cells in TME [43]. Several studies have reported the influence of tumor-associated dendritic cells, macrophages, T cells, and B cells on TME and proposed immunotherapeutic strategies based on tumor-associated macrophages [44], 45]. Dendritic cells, the most powerful antigen-presenting cells, are cellular components of the tumor inflammatory microenvironment that play a crucial role in adaptive immune responses [46]. They can induce specific T lymphocyte responses against tumor-associated antigens. B cells corresponding to T cells, some studies have pointed out that the effect of B cell infiltration of tumor immunotherapy is positively correlated. Tumor-associated macrophages actively contribute to extracellular matrix modification and facilitate the formation of new blood and lymphatic vessels within neoplastic tissues [47]. Immunotherapy targeting tumor-associated macrophages is a highly promising anti-cancer approach. Comparative analysis revealed distinct immune cell infiltration patterns, with B lymphocytes, dendritic cells, macrophages, and T lymphocytes demonstrating differential abundance between malignant and normal tissue specimens. Furthermore, B cells, macrophages, and T cell populations exhibited statistically significant variations when comparing high-risk versus low-risk patient cohorts; however, those of dendritic cells were not different. These results suggest that our prognostic model effectively reflects immune microenvironment dynamics and correlates with patient clinical outcomes.

Furthermore, the expression of the risk score-related genes was positively correlated with sensitivity to commonly used chemotherapeutic drugs. The risk score was significantly correlated with sensitivity to irofulven, indicating that irofulven may be an effective drug for HNSCC. Among these risk score genes, the correlation between PTGR1 and irofulven was most significant. Therefore, we investigate the relationship between PTGR1 and ferroptosis in HNSCC. PTGR1 knockdown elevated ferroptosis levels in HNSCC cells, suggesting that PTGR1 regulates ferroptosis in this malignancy. Besides, the above resultsIndicatethe significant correlation between irofulven and PTGR1. We hypothesized that irofulven could influence the ferroptosis of HNSCC through targeting PTGR1, which means the drug irofulven could be combined with ferroptosis inducer RSL3 treat HNSCC through enhancing the ferroptosis levels in HNSCC. And our subsequent experiments verified this. The *in vitro* and *in vivo* experiments showed that irofulven and RSL3 synergistically inhibited the growth of HNSCC. Notably, the mechanism of irofulven regulating ferroptosis in HNSCC is still unclear. We will investigate it in our future work. Altogether, this study offers a reliable theoretical basis for the diagnosis and treatment of HNSCC through targeting ferroptosis.

This study has certain limitations. While we identified DEGs and DMGs between different risk groups in HNSCC sequencing data, which suggest a significant association with HNSCC risk, our primary focus was on the construction and validation of the prognostic risk model. The specific functional roles and biological significance of these DEGs and DMGs in HNSCC pathogenesis warrant further in-depth investigation in subsequent studies. Furthermore, although several ferroptosis-related signatures have been reported in the literature, for instance, three ferroptosis-associated tumor antigens caveolin1 (CAV1), ferritin heavy chain (FTH1), and solute carrier 3A2 (SLC3A2) shown to be overexpressed and mutated in HNSCC, correlating with poor prognosis and antigen-presenting cell infiltration [48], and prognostic ferroptosis-related gene models constructed based on immune subtypes [49]. Our study distinctively concentrated on identifying prognostic genes capable of modulating ferroptosis levels in HNSCC. We further aimed to discover small-molecule compounds that, via these prognostic genes, could influence sensitivity to ferroptosis inducers for potential combination therapy. However, the performance of the constructed LASSO model was suboptimal, indicating a need for refined modeling strategies in the future to enhance its clinical applicability. Additionally, while this work explored potential mechanisms of ferroptosis in HNSCC, it is important to acknowledge that HNSCC involves other regulatory pathways. For example, studies have reported that nicotine-induced cholinergic receptor nicotinic alpha 5 Subunit (CHRNA5) promotes HNSCC recurrence and metastasis via the carboxylesterase 1 (CES1) axis [50], and that plasma prefoldin subunit 2 (PFDN2) suppresses HNSCC progression by limiting CD64 in a monocyte-driven inflammatory microenvironment [51]. These findings highlight the complexity of the regulatory network in HNSCC, necessitating further exploration of the interplay among different mechanistic pathways in future research. Finally, we investigated the combined effect of the small-molecule compound irofulven and the ferroptosis inducer RSL3. Correlation analysis via CellMiner suggested that irofulven may modulate HNSCC sensitivity to RSL3 through PTGS1. Future studies will explore whether irofulven can directly regulate the expression or activity of PTGR1.

## Supplementary Material

Supplementary Material
