# Supplementary material for: Irofulven and RSL3 synergistically target ferroptosis-related gene PTGR1 to treat head and neck squamous cell carcinoma
Source: Open Life Sci. 2026 Jun 11;21(1):20251332. doi: 10.1515/biol-2025-1332 (PMC13267736; doi:10.1515/biol-2025-1332)
Supplement: Supplementary file 1 — Supplementary Material [file j_biol-2025-1332_suppl_001.pdf]

Irofulven and RSL3 Synergistically Target Ferroptosis-related Gene PTGR1 to Treat Head and Neck Squamous Cell Carcinoma

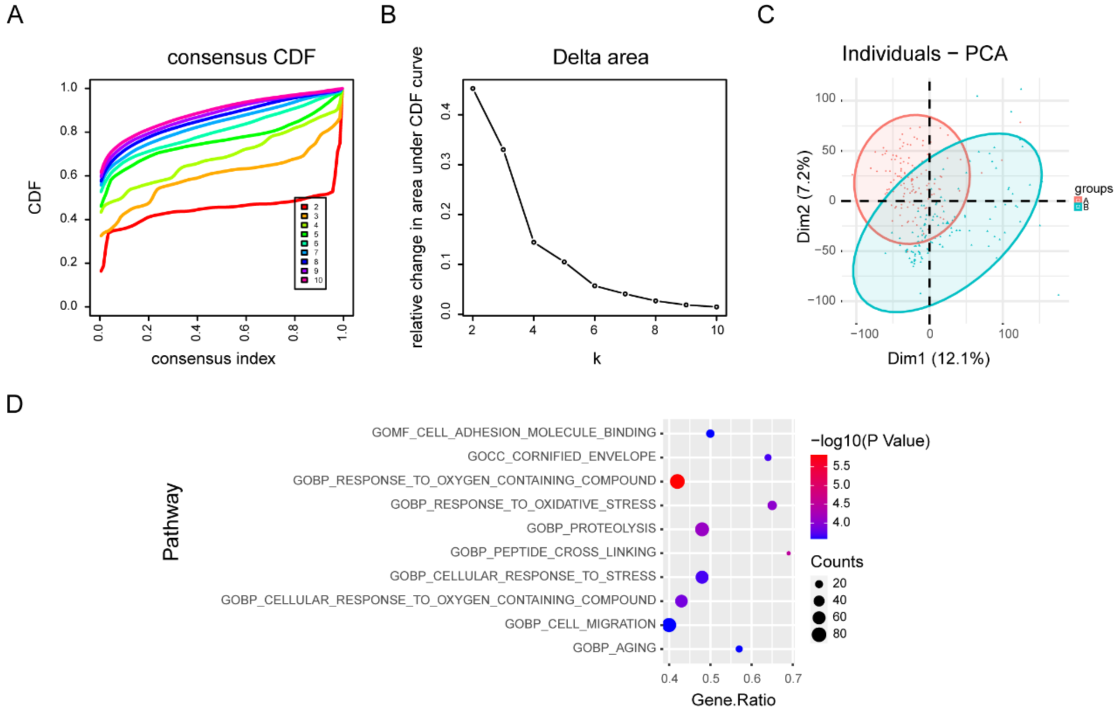

Fig S1. The Consensus clustering analysis of ferroptosis-related genes.

A. CDF graph. **B.** CDF delta area graph. **C.** PCA diagram. **D.** The dotplots of the enriched pathway.

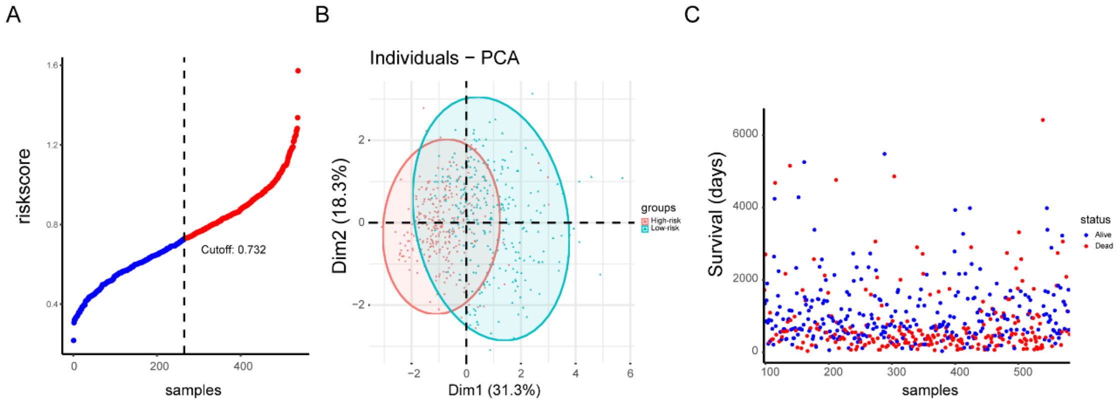

Fig.S2 LASSO regression of HNSCC training set and validation set.

A. Distribution of risk score between low and high-risk groups in the TCGA training set. **B.** Principal component analysis (PCA) to validate screening results. **C.** The

survival status of patients in the TCGA training set.

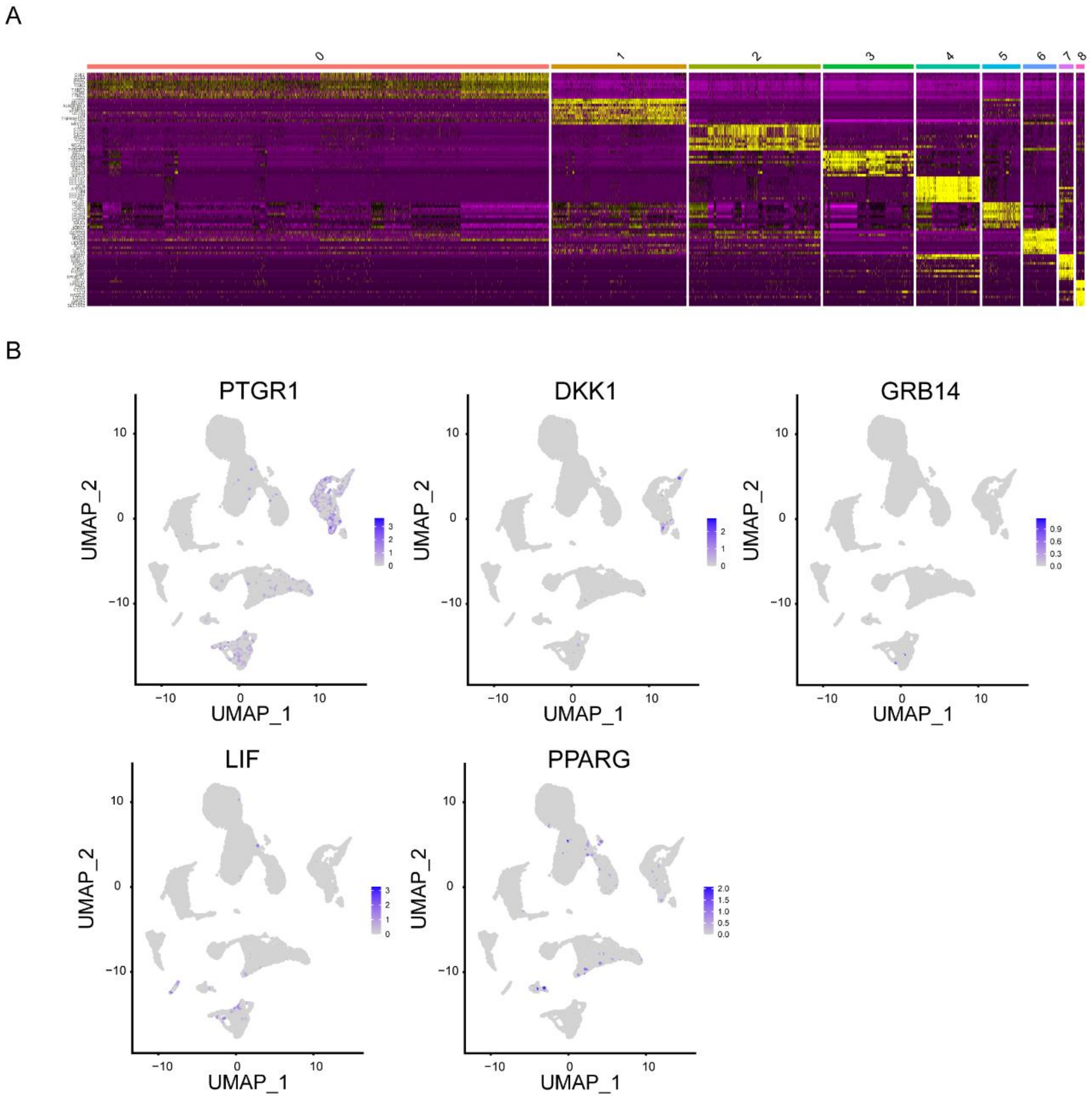

Fig.S3 Single-cell analysis of the risk score of HNSCC.

A. Heatmap showing relative expression of the top 70 genes of each cluster. **B.** Scatter plots of the risk score-related gene expression distribution in 9 clusters. 0, B plasma cells; 1, Dendritic cells; 2, Endothelial cells; 3, Fibroblasts; 4, Macrophages; 5, Malignant cells; 6, Mast cells; 7, Myocytes; 8, T cells.

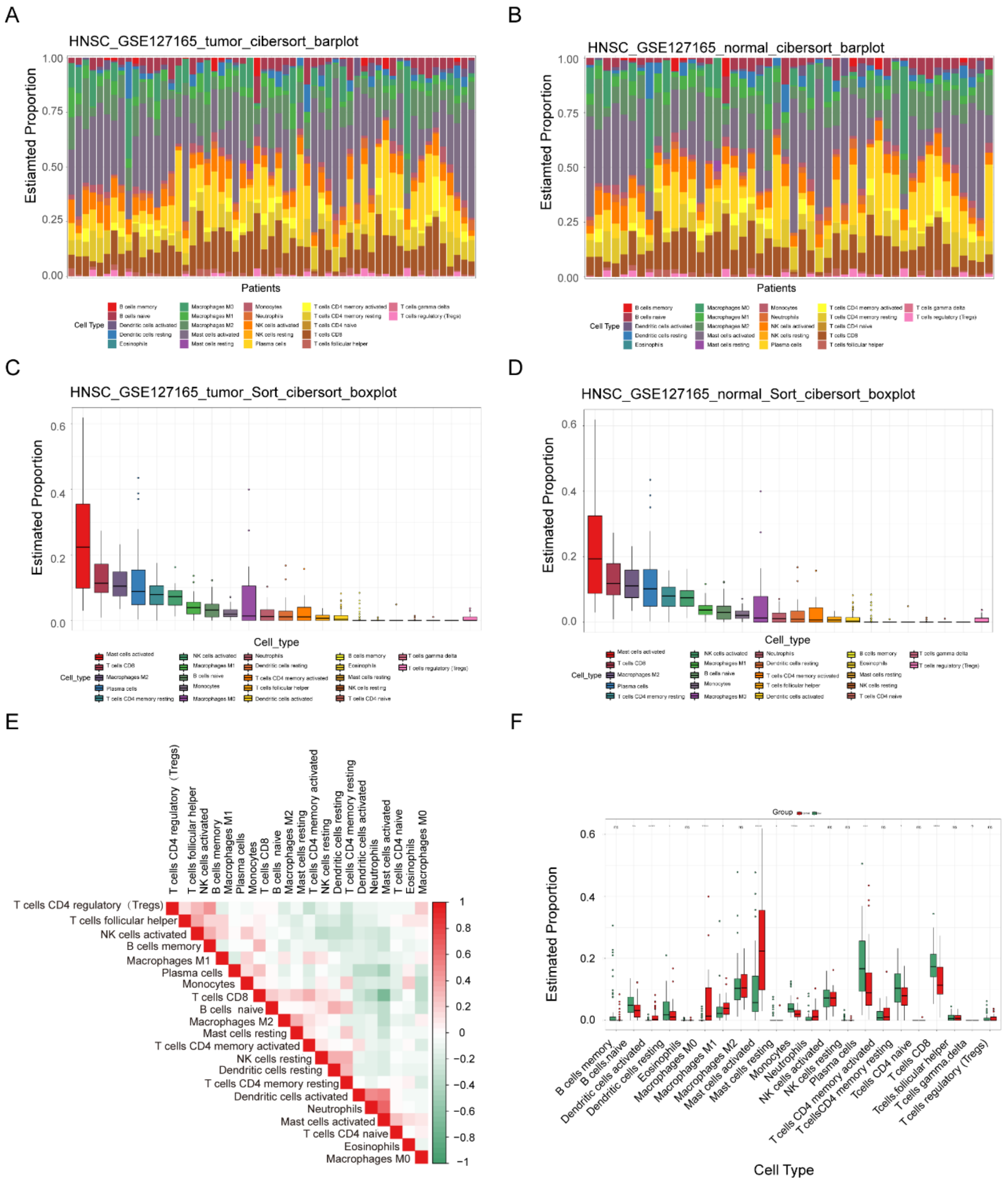

Fig.S4 The immune infiltration of 22 immune cell types in normal and tumor groups.

A. The mean proportion of 22 immune cell types in the tumor group. **B.** The mean proportion of 22 immune cell types in the normal group. **C.** The boxplot of the enriched proportion of 22 immune cell types in the tumor group. **D.** The boxplot of the enriched proportion of 22 immune cell types in the normal group. **E.** Correlation matrix of all 22 immune cell proportions. **F.** The differentiation of 22 immune cell types between the tumor and normal groups.

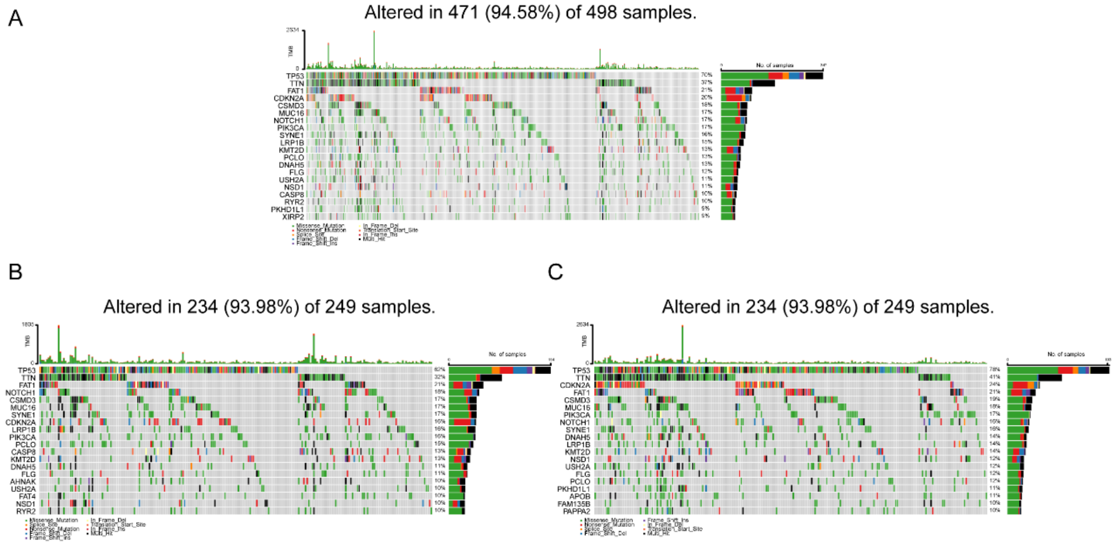

Fig.S5 Top genes harbored somatic variants.

A-C. Occurrence of the top 20 somatically mutated genes in all patients (A), the low-risk group (B), and the high-risk group (C) separately.
